# A high energy density asymmetric supercapacitor utilizing a nickel phosphate/graphene foam composite as the cathode and carbonized iron cations adsorbed onto polyaniline as the anode†

**DOI:** 10.1039/c7ra12028a

**Published:** 2018-03-26

**Authors:** A. A. Mirghni, M. J. Madito, K. O. Oyedotun, T. M. Masikhwa, N. M. Ndiaye, Sekhar. J. Ray, N. Manyala

**Affiliations:** Department of Physics, Institute of Applied Materials, SARCHI Chair in Carbon Technology and Materials, University of Pretoria Pretoria 0028 South Africa ncholu.manyala@up.ac.za +(27)12 420 2516 +(27)12 420 3549; Department of Physics, College of Science, Engineering and Technology, University of South Africa Private Bag X6, Florida, 1710, Science Campus, Christiaan de Wet and Pioneer Avenue, Florida Park Johannesburg 1710 South Africa

## Abstract

This work presents the effect of different contents of graphene foam (GF) on the electrochemical capacitance of nickel phosphate Ni_3_(PO_4_)_2_ nano-rods as an electrode material for hybrid electrochemical energy storage device applications. Pristine Ni_3_(PO_4_)_2_ nano-rods and Ni_3_(PO_4_)_2_/GF composites with different GF mass loadings of 30, 60, 90 and 120 mg were synthesised *via* a hydrothermal method. The electrochemical behavior of pristine Ni_3_(PO_4_)_2_ and Ni_3_(PO_4_)_2_/GF composites were analysed in a three-electrode cell configuration using 6 M KOH electrolyte. The Ni_3_(PO_4_)_2_/90 mg GF composite sample exhibited the highest specific capacity of 48 mA h g^−1^ at a current density of 0.5 A g^−1^. The electrochemical behavior of the Ni_3_(PO_4_)_2_/90 mg GF composite was further analysed in a two-electrode hybrid asymmetric device. A hybrid asymmetric device was fabricated with Ni_3_(PO_4_)_2_/90 mg GF as the cathode and carbonized iron cations (Fe^3+^) adsorbed onto polyaniline (PANI) (C-FP) as the anode material (Ni_3_(PO_4_)_2_/90 mg GF//C-FP) and tested in a wide potential window range of 0.0–1.6 V using 6 M KOH. This hybrid device achieved maximum energy and power densities of 49 W h kg^−1^ and 499 W kg^−1^, respectively, at 0.5 A g^−1^ and had long-term cycling stability.

## Abstract 2

This work presents the effect of different contents of graphene foam (GF) on the electrochemical capacitance of nickel phosphate Ni_3_(PO_4_)_2_ nano-rods as an electrode material for hybrid electrochemical energy storage device applications.

## Graphical abstract

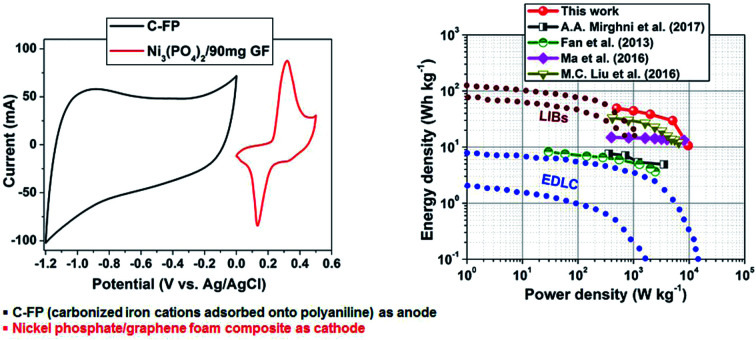

## 1. Introduction

Over the past years, supercapacitors have attracted much attention, because of their long cycle life, short charging time, high power density, *etc.*, and they are used in a variety of domestic, commercial and industrial applications such as portable electronic devices, micro-electromechanical systems, and hybrid electric/plug-in-hybrid vehicles.^1–4^ Nowadays, amongst other energy storage systems, supercapacitors have become the main focus for renewable and sustainable energy sources.^5–9^ Therefore, for future energy storage systems, effort is focussed on developing new materials with high electrochemical performance.^7,9^ Supercapacitors compared to rechargeable batteries deliver high power density at a very fast rate, but suffer from low energy density, on the other hand, batteries deliver high energy density, but suffer from low power density and as a result supercapacitors and batteries complement each other in many applications.^6,7,10^ Depending on the energy storage mechanism, electrochemical capacitors can be divided into two types of capacitors, namely, electric double layer capacitors (EDLCs) and pseudo-capacitors.^7^ Carbon materials with a high surface area such as carbon nanotubes, graphene, and porous carbon exhibit EDLC behavior which stores charges at the interface between the electrode and the electrolyte. The large-scale application of EDLCs is limited by their low specific capacitance (100–200 F g^−1^).^11,12^ In contrast, transition-metal oxides such as RuO_2_, MnO_2_, *etc.* and/or conducting polymers such as PANI, PPy, PEDOT, *etc.*, exhibit predominantly pseudocapacitive behavior.^7,13,14^ In the pseudocapacitor, energy is predominantly stored in reversible faradic redox reactions occurring on or near the electrode surface and result in a specific capacitance that is 10–100 times higher than that of EDLCs.^7,15^ However, the self-high electrical resistance of transition-metal oxides limits the charge–discharge rate, energy density and power density of the electrode materials, which are considered as the main factors for strong-willed energy storage applications.

As inexpensive and electrochemical active material, transition-metal phosphate such as Mn_3_(PO_4_)_2_, BiPO_4_, Co_2_P_2_O_7_, VOPO_4_, and Ni_2_P_2_O_7_ attracted the attention of the researchers and exhibited a potential interest in energy storage applications. Among these metal phosphates, nickel phosphate is used in different fields due to the physio-chemical properties, for instance in electro-catalyst for the H_2_ evolution, catalysts for organic synthesis, and anode materials for lithium-ion batteries.^16–18^ Few studies on nickel phosphate as an electrode in supercapacitor applications were recently reported. For instance, Huan *et al.* synthesized Ni_11_(HPO_3_)_8_(OH)_6_ under the hydrothermal condition and reported a high specific capacitance of 295 F g^−1^ (32 mA h g^−1^) at a current density of 0.625 A g^−1^ in a 3.0 M KOH electrolyte.^19^ Manickam *et al.* synthesized NaNiPO_4_ using the facile hydrothermal method and obtained a high specific capacitance of 125 F g^−1^ (4.17 mA h g^−1^) at a current density of 1.0 A g^−1^ in a 2.0 M KOH.^20^ Ni_3_(PO_4_)_2_ can be synthesized by different methods, but the hydrothermal method is a facile and rapid method that can offer unique morphologies, energy saving, simplicity, high purity and crystalline material.^20^

Similar to transition-metal oxides, nickel phosphate suffers from slow ion transfer rate which increases the electrical resistance of the material and limits charge/discharge ability of the electrode material. Now, to overcome the drawback in the electrical resistance of the nickel phosphate, graphene is an outstanding candidate to increase the electrical conductivity of the electrode material. In fact, graphene has been found to exhibit exceptionally high thermal conductivity, electrical conductivity, strength, the high specific surface area up to 2675 m^2^ g^−1^ and the intrinsic capacitance of graphene was recently found to be 21 μF cm^−2^.^21–24^ These studies assert that graphene is the ideal material for increasing the conductivity and specific surface area of the electrode materials by surface coating. Therefore, the addition of graphene into Ni_3_(PO_4_)_2_ is expected to improve the electrochemical performance of the Ni_3_(PO_4_)_2_ electrode for supercapacitor applications.

Herein, we report the synthesis of Ni_3_(PO_4_)_2_ and Ni_3_(PO_4_)_2_/graphene foam (GF) composites nanostructures by a simplistic and eco-friendly hydrothermal technique. The GF with different mass loading in the range of 30 to 120 mg/100 mL of deionized water were added into Ni_3_(PO_4_)_2_ synthesis to improve the electrochemical performance of the pristine Ni_3_(PO_4_)_2_ electrode. The Ni_3_(PO_4_)_2_/90 mg GF composite revealed a highest specific capacity of 48 mA h g^−1^, as compared to the pristine Ni_3_(PO_4_)_2_ which exhibited a specific capacity of 17 mA h g^−1^ at a current density of 0.5 A g^−1^ in 6 M KOH. To evaluate the practical application of the Ni_3_(PO_4_)_2_/90 mg GF composite, a two-electrode hybrid cell device was fabricated with Ni_3_(PO_4_)_2_/90 mg GF composite served as a positive electrode and carbonized iron cations (Fe^3+^) adsorbed onto polyaniline (PANI) (C-FP) as a negative electrode. The Ni_3_(PO_4_)_2_/90 mg GF//C-FP hybrid device was found to perform at a high cell voltage of 1.6 V in 6 M KOH. This device exhibited a specific capacity of 48 mA h g^−1^ at a current density of 0.5 A g^−1^ with a maximum energy and power densities of 49.2 W h kg^−1^ and 499 W kg^−1^ respectively. In addition, a Ni_3_(PO_4_)_2_/90 mg GF//C-FP device showed excellent cycling stability with 53% capacity retention over 10 000 galvanostatic charge–discharge cycles at a current density of 10 A g^−1^.

## 2. Experimental details

### 2.1. Materials

Nickel nitrate (NiN_2_O_6_·6H_2_O) and ammonium phosphate (N_2_H_9_PO_4_, purity 98%), were purchased from Sigma Aldrich. Polycrystalline Ni foam 3D scaffold template with an areal density of 420 g m^2^ and thickness of 1.6 mm was purchased from Alantum (Munich, Germany). Potassium hydroxide (KOH, min 85%) was purchased from Merck (South Africa).

### 2.2. Synthesis of Ni_3_(PO_4_)_2_ using hydrothermal method

**Scheme 1:**
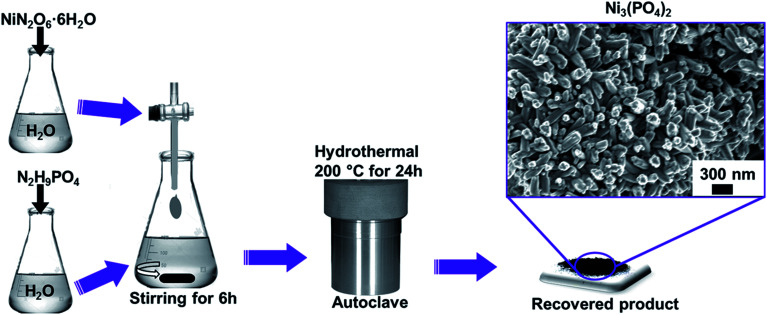
Preparation procedure of nanostructured nickel phosphate.

All the reagents used in the experiment are of analytical grade and were used as received without further purification. Typically, NiN_2_O_6_·6H_2_O (1.745 g) and N_2_H_9_PO_4_ (0.264 g) were used as precursors for the synthesis of nickel phosphate. The masses were corresponding to the equivalent molar ratios of Ni_3_(PO_4_)_2_. Each precursor was completely dissolved in 50 mL of deionized water, as demonstrated by Scheme 1. Then, the solution of NiN_2_O_6_·6H_2_O was added dropwise into the solution of N_2_H_9_PO_4_ and stirred for 6 h. The resultant solution was transferred into a sealed, Teflon-lined, stainless-steel autoclave and kept at a temperature of 200 °C for 24 h and then cooled down to the room temperature. Subsequently, the obtained precipitate was filtered and washed with deionized water several times and dried at 60 °C overnight. Finally, the recovered product of Ni_3_(PO_4_)_2_ was obtained which showed compact nano-rods morphology as displayed by micrograph in Scheme 1.

### 2.3. Synthesis of Ni_3_(PO_4_)_2_/GF composites using hydrothermal method

The graphene foam (GF) were prepared according to our recent work.^25,26^ Briefly, the Ni foam (NF) was placed at a centre of a CVD quartz tube. Before graphene growth, the nickel foam was annealed at 1000 °C under a mixture of argon (Ar) and hydrogen (H_2_) gasses for 60 min. Subsequently, methane (CH_4_) gas (acting as a carbon source) was introduced into the reaction chamber at 1000 °C for 10 min and the flow rates of the gasses Ar : H_2_ : CH_4_ were 300 : 200 : 10 sccm respectively. After graphene growth, the samples were rapidly cooled by pushing the quartz tube to a cooler region of the furnace. In order to obtain the GF, the samples were further dipped in 3.0 M HCl at 80 °C to ensure complete etching of the nickel supporting structure. After complete etching of the nickel template, the recovered GF was washed several times with deionized water and dried at 60 °C. Thereafter, the GF was crushed into powder and sonicated.

The Ni_3_(PO_4_)_2_/GF composite was prepared using a hydrothermal technique following the same procedure shown in Scheme 1 for pristine synthesis. Typically, 30 mg of the as-prepared powdered GF was dispersed in 100 mL of deionized water and taken through ultra-sonication for several hours until the solution became homogeneous. Then, 1.745 g of NiN_2_O_6_·6H_2_O and 0.264 g of N_2_H_9_PO_4_ were added to the sonicated GF solution and the entire mixture was further stirred for 6 h. Thereafter, the mixture was transferred into a sealed, Teflon-lined, stainless-steel autoclave (120 mL) and kept at a temperature of 200 °C for 24 h. After cooling to room temperature, the recovered product of Ni_3_(PO_4_)_2_/30 mg GF composite was washed with deionized water and dried overnight at 60 °C. This procedure was repeated for the synthesis of Ni_3_(PO_4_)_2_/60 mg GF, Ni_3_(PO_4_)_2_/90 mg GF and Ni_3_(PO_4_)_2_/120 mg GF composites using 60 mg, 90 mg and 120 mg GF respectively.

### 2.4. Structural, morphological and composition characterization

The crystallite structure analysis of pristine Ni_3_(PO_4_)_2_ and Ni_3_(PO_4_)_2_/GF composites was carried out using X-ray diffraction (XRD) XPERTPRO diffractometer (PANalytical BV, Netherlands) with theta/2 theta geometry, operating with a cobalt (Co) tube at 50 kV and 30 mA. A T64000 micro-Raman spectrometer (HORIBA Scientific, Jobin Yvon Technology) with a 514 nm laser wavelength and spectral acquisition time of 120 s was used to characterize the pristine Ni_3_(PO_4_)_2_ and Ni_3_(PO_4_)_2_/GF composites. The Raman system laser power was set as low as 3 mW in order to minimize heating effects. X-ray photoelectron spectroscopy (XPS) measurements of the samples were conducted using a Physical Electronics VersaProbe 5000 spectrometer operating with a 100 μm monochromatic Al-Kα exciting source. The scanning electron microscopy (SEM) images were obtained using a Zeiss Ultra Plus 55 field emission scanning electron microscope (FE-SEM) operated at 2.0 kV to obtain the morphology of the pristine Ni_3_(PO_4_)_2_ and Ni_3_(PO_4_)_2_/GF composites. For high-resolution transmission electron microscopy (HRTEM) analysis the ethanol solution containing the samples was dispersed on a formal-coated copper grid and the analysis was carried out in a Jeol JEM-2100F Field Emission Electron Microscope with a maximum analytical resolution of 200 kV and a probe size of <0.5 nm.

### 2.5. Electrochemical characterization

All electrochemical analysis were carried out on a Biologic VMP-300 potentiostat (Knoxville TN 37,930, USA) controlled by the EC-Lab V10.37 software at room temperature. In the three-electrode system, a glassy carbon plate was used as the counter electrode and Ag/AgCl (3 M KCl) electrode served as the reference electrode. The working electrode was prepared by coating a mixture of 80 wt% active material, 10 wt% carbon black and 10 wt% polyvinylidene fluoride (PVDF) binder dispersed in *N*-methylpyrrolidone (NMP)solution onto a piece of nickel foam (1 × 1 cm^2^). After coating, the as-prepared electrode was dried at 60 °C overnight, and thereafter, the coated active material was pressed onto the nickel foam under a pressure of 30 MPa. The mass loading of active material in working electrode was 2.0 mg. The electrochemical measurements were carried out in the three-electrode system in 6 M KOH aqueous electrolyte solution. Cyclic voltammetry (CV) was performed at scan rates of 5 to 100 mV s^−1^ in the voltage range of 0.0–0.5 V *vs.* Ag/AgCl. The galvanostatic charge/discharge (GCD) curves were tested at current densities ranging from 0.5 A g^−1^ to 10 A g^−1^ in the voltage range of 0.0–0.4 V *vs.* Ag/AgCl. The electrochemical impedance spectroscopy (EIS) was measured in an open circuit potential over a frequency range of 10 mHz to 100 kHz. The electrochemical analysis of the active material was also evaluated in a two-electrode hybrid cell device. In the two-electrode hybrid cell device, Ni_3_(PO_4_)_2_/90 mg GF (2.6 mg cm^−2^) electrode served as a positive electrode, while the C-FP (1.4 mg cm^−2^) material was selected as the negative electrode, amounting to a total mass loading estimated as 4.0 mg cm^−2^ for both active materials in the hybrid device. The negative electrode material was synthesized by pyrolysis of the iron-containing mixture coated on nickel foam (current collector) in a tube furnace under the N_2_ atmosphere at 850 °C for 2 h as reported in our earlier publication.^27^ Briefly, iron nitrate nonahydrate (0.2 g) and PANI (0.0125 g) were dissolved in ethanol (50 mL) under continuous stirring until the ethanol was almost completely evaporated. Thereafter, the moist mixture was coated on nickel foam which was loaded in a tube furnace and heated to 850 °C and pyrolyzed for 2 h under the N_2_ atmosphere. After pyrolysis, the as-prepared sample was named as C-FP (see ESI for more details†).

The specific capacity, *Q*_s_ (mA h g^−1^) of the materials using GCD curves were calculated using the following expression:^27^1
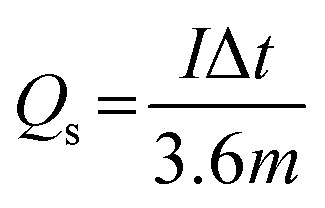
where *I* is the discharge current in mA, *m* is the mass loading of the electrode in mg, Δ*t* is the time in seconds taken for a complete discharge cycle.

The energy density *E*_d_ (W h kg^−1^) and power density *P*_d_ (W kg^−1^) of asymmetric hybrid device were calculated from the CD curves using the following equations:2
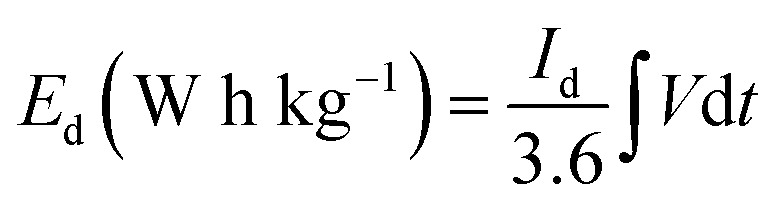
3
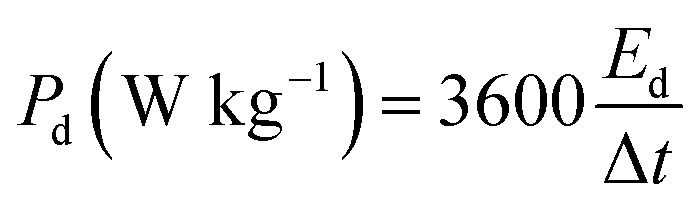
Where ∫*V*d*t* is the area under the discharge curve, *I*_d_ is the current density (A g^−1^), *V* is the potential window (V), and Δ*t* is the discharge time (s).

## 3. Results and discussion

### 3.1. Structural, composition, and morphological characterization

**Fig. 1:**
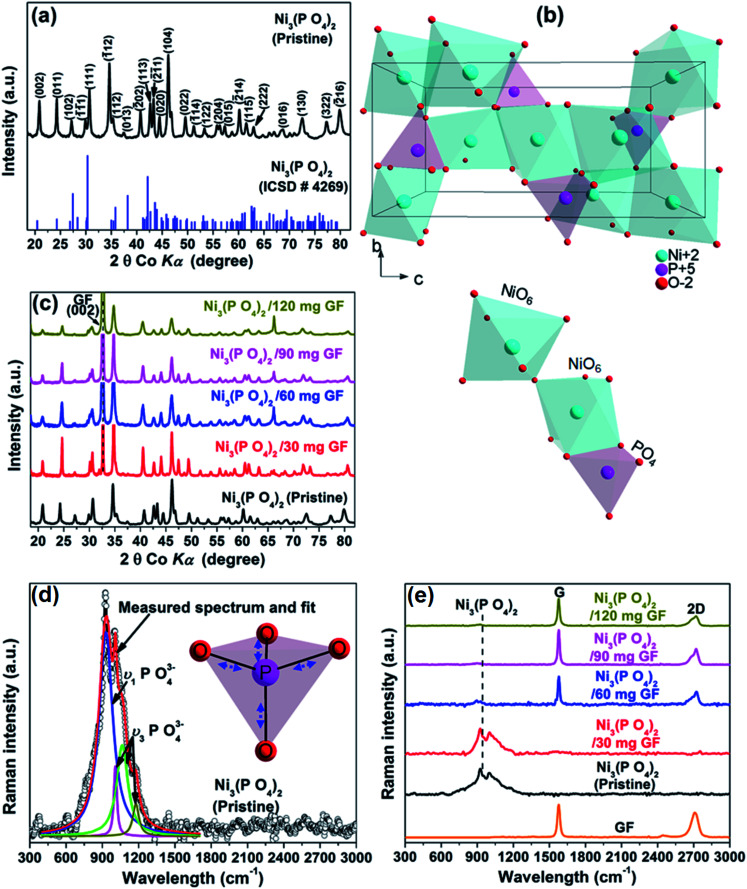
(a) The X-ray diffraction of the pristine Ni_3_(PO_4_)_2_ sample and the matching ICSD card no. 4269, (b) a view of the crystal structure of Ni_3_(PO_4_)_2_ as projected in the (b and c) plane based on Crystallographic Information File (CIF) of ICSD card no. 4269 and (c) the X-ray diffraction of the pristine Ni_3_(PO_4_)_2_ and Ni_3_(PO_4_)_2_/GF composites at different GF mass loading, (d) the Raman spectrum of the pristine Ni_3_(PO_4_)_2_ sample and the Lorentz fittings of peaks at 937, 1000, 1050 and 1164 cm^−1^ (the insert figure displays a view of vibrational (stretching) modes of the [PO_4_] tetrahedron) and (e) the Raman spectra of the pristine Ni_3_(PO_4_)_2_, Ni_3_(PO_4_)_2_/GF composites at different GF mass loading and that of GF.

Fig. 1(a) shows the XRD of the pristine nickel phosphate sample which displays well-defined crystalline peaks and these peaks were indexed using the matching Inorganic Crystal Structure Data-base (ICSD) card no. 4269 with chemical formula Ni_3_(PO_4_)_2_, crystal system monoclinic, space-group *P*121/*c*1, and cell parameters: *a* = 5.830 Å, *b* = 4.700 Å, *c* = 10.107 Å, *β* = 91.22° and *Z* = 2. Fig. 1(b) shows the crystal structure of Ni_3_(PO_4_)_2_ as projected in the (*b* and *c*) plane based on Crystallographic Information File (CIF) of ICSD card no. 4269. In this structure, the Ni ions which appear in two types of sites of the structure are octahedrally coordinated by oxygen (O) ions with average Ni–O bond lengths of 2.081 and 2.067 Å, and phosphorus (P) atoms are tetrahedrally coordinated by O with average P–O bond length of 1.547 Å.^28^ Every [NiO_6_] octahedron is connected to another [NiO_6_] by common oxygen edge (O_5_Ni–O–NiO_5_) and to [PO_4_] tetrahedron by two common oxygen edges. Therefore, this would suggest that the O–O repulsion might be responsible for the weakening of the [NiO_6_]–[PO_4_] formation and as a result making a Ni_3_(PO_4_)_2_ structure chemically less stable. Nonetheless, the strong P–O covalent bonds make the Ni_3_(PO_4_)_2_ structure chemically very stable. In brief, the structure of the pristine sample can be described as a three-dimensional network built up from corners sharing of [NiO_6_]–[NiO_6_]–[PO_4_]. Fig. 1(c) shows the XRD of the pristine Ni_3_(PO_4_)_2_ and Ni_3_(PO_4_)_2_/GF composites at different GF mass loading. It can be seen that GF mass loading does not alter the XRD pattern of the nickel phosphate. A (002) diffraction peak which corresponds to GF confirms the presence of GF in the composites.

Fig. 1(d) shows the Raman spectrum of the pristine nickel phosphate sample which displays the band at about 940 cm^−1^ and this band was deconvoluted with Lorentz fitting of peaks at 937, 1000, 1050 and 1164 cm^−1^ assigned to the symmetric stretching mode, *ν*_1_ (937 cm^−1^), and antisymmetric stretching modes, *ν*_3_ (1000, 1050 and 1164 cm^−1^) of phosphate oxyanions.^29,30^ The insert to Fig. 1(d) displays a view of vibrational (stretching) modes of [PO_4_] tetrahedron which shows that all of the oxygen's coordinating the phosphorus can stretch away from it at the same time (symmetric stretching mode, *ν*_1_), or two can stretch while the other two contract (antisymmetric stretching mode, *ν*_3_). The Raman spectrum of the pristine nickel phosphate sample does not show any obvious modes originating from [NiO_6_] octahedron. Fig. 1(e) shows the Raman spectra of the pristine Ni_3_(PO_4_)_2_, Ni_3_(PO_4_)_2_/GF composites at different GF mass loading and that of GF. The as-prepared GF shows a typical Raman spectrum of graphene which depicts G-mode at 1570 cm^−1^ and 2D-mode at 2710 cm^−1^.^31^ In Fig. 1(e), Ni_3_(PO_4_)_2_/30 mg GF composite shows Raman band at about 940 cm^−1^ originating from nickel phosphate, however, does not show modes of GF. Nevertheless, at higher GF mass loading (*i.e.* Ni_3_(PO_4_)_2_/60 mg GF, Ni_3_(PO_4_)_2_/90 mg GF and Ni_3_(PO_4_)_2_/120 mg GF composites) the G and D bands of GF are noticeable, but that of nickel phosphate is not observed and that could be as a result of GF adhering to the surface of the nickel phosphate.

**Fig. 2:**
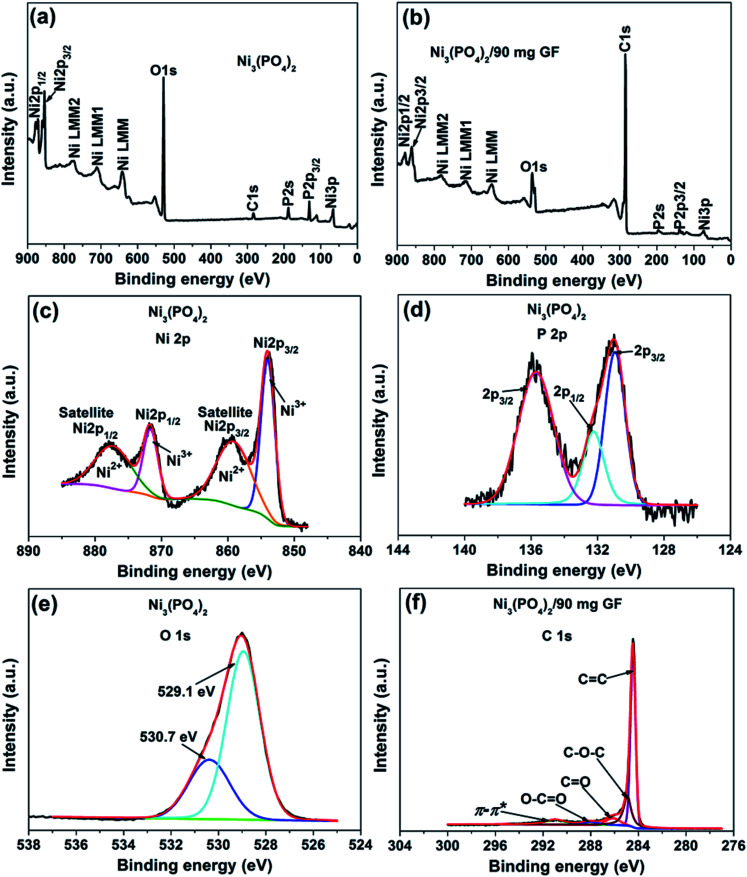
(a) and (b) The wide scan XPS spectra of the as-received pristine nickel phosphate sample and the Ni_3_(PO_4_)_2_/90 mg GF composite respectively. The core level spectrum of (c) Ni 2p, (d) P 2p and (e) O 1s of a pristine sample. (f) The core level spectrum of C 1s of a composite sample.

The surface chemistry of the pristine nickel phosphate sample and the Ni_3_(PO_4_)_2_/90 mg GF composite was analyzed by XPS. Fig. 2(a) and (b) show the wide scan XPS spectra of the as-received (*i.e.*, without sputter cleaning) pristine nickel phosphate sample and the Ni_3_(PO_4_)_2_/90 mg GF composite chosen because it showed a good electrochemical results that will be discussed below respectively, which displays the main elements (Ni 2p, P 2p, O 1s and C 1s) of the composition of the samples. A pristine sample shows 15.25 at% of Ni, 14.10 at% P, and 70.65 at% O suggesting that a sample is predominantly composed of oxygen. On the other hand, a composite sample shows 1.66 at% of Ni, 0.37 at% P, 13.61 at% O and 84.36 at% C. A high concentration of C suggests that a composite surface is dominantly coated by C which makes the surface concentration of the main elements (Ni 2p, P 2p and O 1s) to appear low since these concentrations are fractional concentrations and the XPS technique is surface sensitive thus focus analysis within the topmost (∼5) atomic layers. Interestingly, a surface of a composite sample can be expected to have a good electrical conductivity owing to the outstanding electrical conductivity of graphene, unlike that of a pristine sample which is predominantly oxygen. Furthermore, the core level spectrum of Ni 2p of a pristine sample reveals the binding energy peaks at 853.7, 859.8, 871.6 and 877.7 eV which agree with Ni 2p_3/2_ and Ni 2p_1/2_, assigned to the two spin–orbit doublets characteristic of Ni^2+^ and Ni^3+^ and the two shakeup satellite,^32,33^ as shown in Fig. 2(c). The core level spectrum of P 2p reveals the binding energy peaks at 131.1 eV and 135.7 eV which were fitted to 2p_1/2_ and 2p_3/2_ doublets (131.1 eV peak) and 2p_1/2_ (135.7 eV peak), as shown in Fig. 2(d). The 2p_1/2_ and 2p_3/2_ doublets (131.1 eV peak) components correspond to the P–P bond, while the 2p_1/2_ at 135.7 eV peak is ascribed to the P–O bond.^34^Fig. 2(e) shows the core level spectrum of O 1s with fitted peaks at 529.1 and 530.7 eV which could be ascribed to O 1s in Ni–O and P–O compounds. Fig. 2(f) shows the core level spectrum of C 1s of a Ni_3_(PO_4_)_2_/90 mg GF composite sample. The fitted binding energy peaks 284.4, 285.0, 286.4, 288.4 and 291.2 eV correspond to sp^2^ C=C (graphene component), C–O–C, C=O, O–C=O (oxide components) and π–π* (satellite peak/electrons transition) bonding respectively.^35–37^ In Fig. 2(f), all fitted peaks reveal the sp^2^ hybridization property of GF, traces of oxygen content present in GF and the π–π* electrons transition which enhances the carbon to carbon bonds in graphene and confirms the high quality of GF.^36–38^ It is worth mentioning that GF (Fig. 2(f)) does not reveal obvious reaction with nickel phosphate, however, it shows formation of high-quality graphene. Consequently, GF can be viewed as a sheet coating the active surface of nickel phosphate in composite samples.

**Fig. 3:**
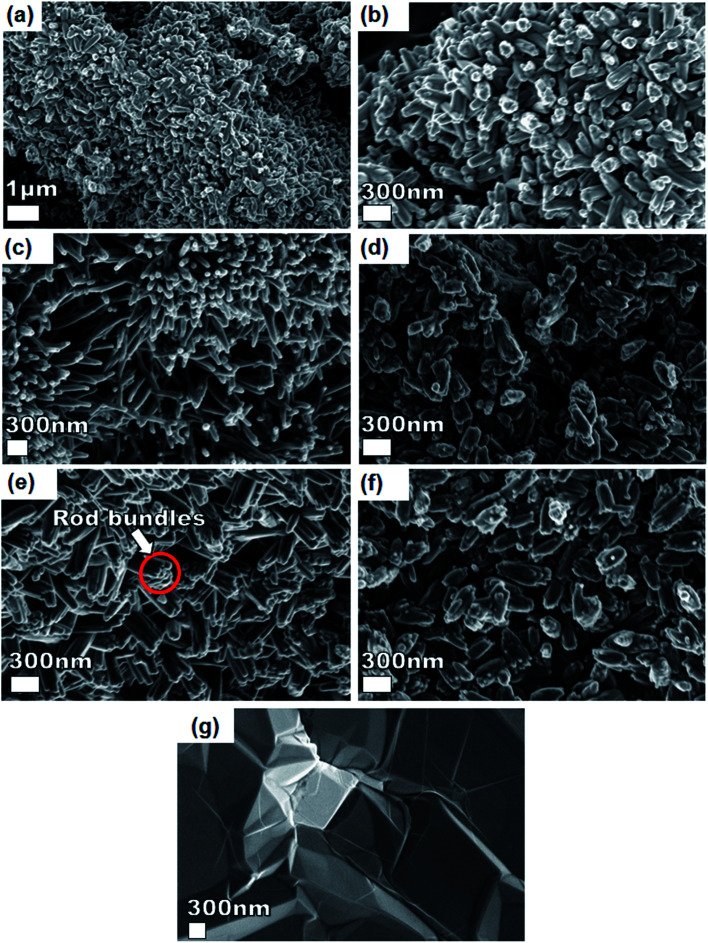
SEM micrographs of (a and b) pristine Ni_3_(PO_4_)_2_ sample at low and high magnification, (c) Ni_3_(PO_4_)_2_/30 mg GF, (d) Ni_3_(PO_4_)_2_/60 mg GF, (e) Ni_3_(PO_4_)_2_/90 mg GF, (f) Ni_3_(PO_4_)_2_/120 mg GF and (g) SEM micrograph of a GF sheet before been crushed into powder and sonicated.

Fig. 3 shows SEM micrographs of the nanostructured pristine Ni_3_(PO_4_)_2_, Ni_3_(PO_4_)_2_/GF composites at different GF mass loading and GF. A pristine Ni_3_(PO_4_)_2_ sample (Fig. 3(a and b)) at low and high magnifications shows nanostructure morphology with the structure becoming clearer (*i.e.*, nano-rods) with an addition of 30 mg GF, as shown by Fig. 3(c) of Ni_3_(PO_4_)_2_/30 mg GF composite. Interestingly, as GF mass loading further increases to 90 mg the observed nano-rods form bundles-like as shown in Fig. 3(e) of Ni_3_(PO_4_)_2_/90 mg GF composite, however, a further increase in GF mass loading up to 120 mg shows poorly defined nanostructures (see Fig. 3(f) of Ni_3_(PO_4_)_2_/120 mg GF composite), which suggest that nano-rods bundles-like morphology observed for Ni_3_(PO_4_)_2_/90 mg GF composite is distorted. It is worth mentioning that the GF which shows sheet-like morphology (see Fig. 3(g)) cannot be identified in the SEM images of the composites as in our recent work.^25^ This could suggest that the as-prepared powdered GF is incorporated (or coated) in the surface of nickel phosphate nanostructures due to ultra-sonication for several hours after been dispersed in deionized water and also after the nickel phosphate precursors were added to it. Nonetheless, from XPS results it was revealed that a high-quality GF is coated on the surface of Ni_3_(PO_4_)_2_.

**Fig. 4:**
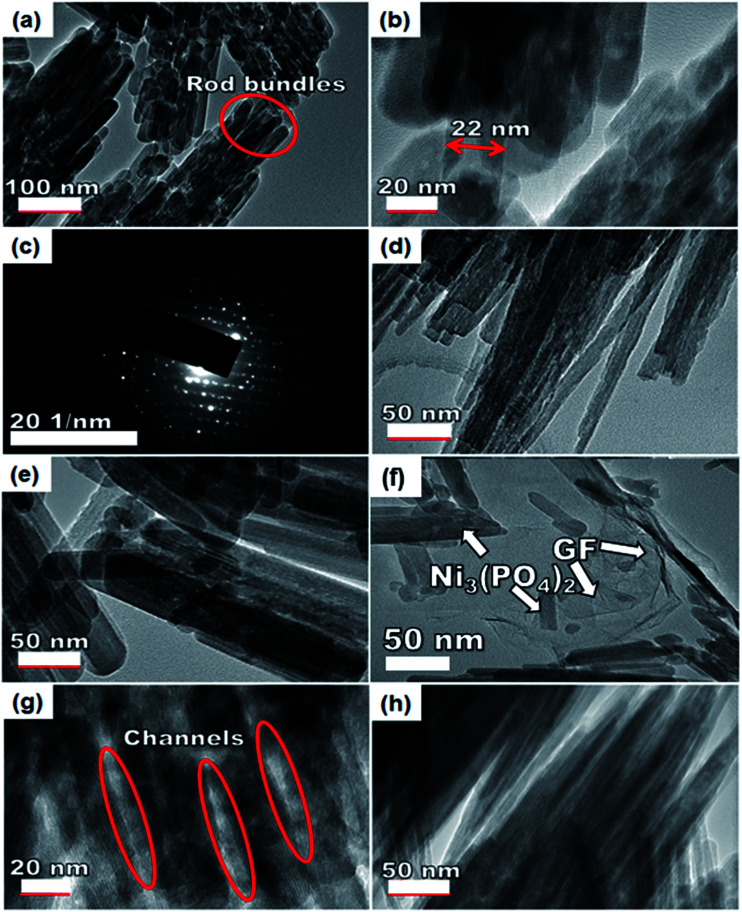
An HRTEM image of the (a and b) pristine Ni_3_(PO_4_)_2_ sample at low and high magnification and the corresponding (c) SAED image of the pristine sample, (d) Ni_3_(PO_4_)_2_/30 mg GF, (e) Ni_3_(PO_4_)_2_/60 mg GF, (f) Ni_3_(PO_4_)_2_/90 mg GF, (g) the channel-like structures of Ni_3_(PO_4_)_2_/90 mg GF and (h) Ni_3_(PO_4_)_2_/120 mg GF.

To further get information on the dispersion of GF in the composites, the nanostructure morphology of pristine Ni_3_(PO_4_)_2_ and Ni_3_(PO_4_)_2_/GF composites at different GF mass loading was further examined by HRTEM and the micrographs are displayed in Fig. 4. Fig. 4(a) and (b) show low and high-resolution HRTEM micrographs of pristine Ni_3_(PO_4_)_2_ respectively. In both micrographs, the results show nano-rods bundles with single rods having an average thickness of about 22 nm, as shown in Fig. 4(b). The crystallinity of the pristine Ni_3_(PO_4_)_2_ was further confirmed by selected area electron diffraction (SAED) pattern, as shown in Fig. 4(c). This pattern shows clear diffraction spots (rings) and this confirms the polycrystalline nature of the material which is in agreement with the XRD results. Fig. 4(d) and (e) show HRTEM micrographs of Ni_3_(PO_4_)_2_/30 mg GF and Ni_3_(PO_4_)_2_/60 mg GF composites respectively. It's clear that as the mass loading of GF increases, the more channel-like structures form within the material up to 90 mg GF mass loading (see Fig. 4(g)), and thereafter, as the GF mass loading increase up to 120 mg the channel-like structures disappear as the nano-rods bundles become more compacted, as shown in Fig. 4(h). This could be due to the high content of GF relative to the Ni_3_(PO_4_)_2_ material in the composite which negatively affects the synergy between the Ni_3_(PO_4_)_2_ and the GF. The GF used in this work is a few-layered graphene with layers number approximately less than five (see Fig. S1†), and it is high-quality graphene as confirmed by XPS analysis.

Nevertheless, with the introduction of graphene foam into the active matrix, the Ni_3_(PO_4_)_2_ nano-rods are seen to effectively attach themselves to the sheet of graphene as shown in Fig. 4(f). A uniform dispersion of the Ni_3_(PO_4_)_2_ nano-rods within the graphene sheet is observed which is energetic for providing the necessary surface required for efficient charge transport and storage, this also confirms a typical sheet-like surface of graphene.

### 3.2. Electrochemical characterization

To study the electrochemical performance of pristine Ni_3_(PO_4_)_2_ and Ni_3_(PO_4_)_2_/GF composite, the three-electrode measurement was carried out. Cyclic voltammetry (CV), charge–discharge (CD), and electrochemical impedance spectroscopy (EIS) for the electrode materials were carried out in 6 M KOH electrolyte. The CV and CD curves analysis of the pristine Ni_3_(PO_4_)_2_ is included in the ESI (Fig. S2†).

**Fig. 5:**
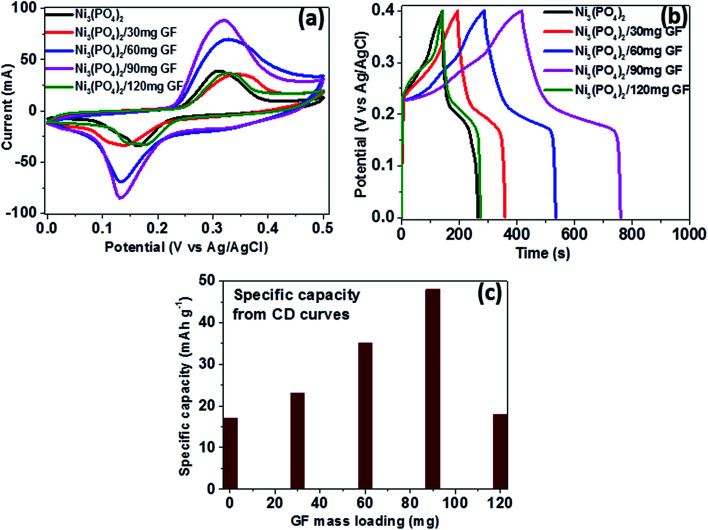
(a) CV curves of pristine Ni_3_(PO_4_)_2_ and Ni_3_(PO_4_)_2_/GF composites at different GF mass loading at a scan rate of 50 mV s^−1^, (b) CD curves of pristine and composites at a current density of 0.5 A g^−1^ within a potential window range of 0.0–0.4 V and (c) specific capacity for pristine and composites calculated from the CD curves in (b).

Fig. 5(a) shows the CV curves of pristine Ni_3_(PO_4_)_2_ and Ni_3_(PO_4_)_2_/GF composites at different GF mass loading at a scan rate of 50 mV s^−1^ in a potential window range of 0.0–0.5 V. In Fig. 5(a), it can be seen that an addition of 30 mg GF to the pristine Ni_3_(PO_4_)_2_ improves the current response of the electrode material. Moreover, for Ni_3_(PO_4_)_2_/60 mg GF composite the current response is found to be further improved compared to Ni_3_(PO_4_)_2_/30 mg GF. A Ni_3_(PO_4_)_2_/90 mg GF composite shows the highest current response suggesting a high specific capacity according to eqn (1), and this could be attributed to the presence of an appropriate amount of GF which effectively synergise with Ni_3_(PO_4_)_2_ and improves its electrical conductivity owing to the outstanding electrical conductivity of graphene*.* A Ni_3_(PO_4_)_2_/120 mg GF composite shows a drop in current response compared to Ni_3_(PO_4_)_2_/90 mg GF composite and that could be due to the high content of GF relative to the Ni_3_(PO_4_)_2_ material in the composite which negatively affects the synergy between the Ni_3_(PO_4_)_2_ and the GF, as mentioned earlier. Fig. 5(b) shows the CD curves of pristine and composites at a current density of 0.5 A g^−1^ within a potential window range of 0.0–0.4 V. In Fig. 5(c) it can be seen that a Ni_3_(PO_4_)_2_/90 mg GF sample shows a considerably longer discharge time than the other composites suggesting a better charge transfer between the composite and the electrolyte which is in agreement with CV curves in Fig. 5(a). Fig. 5(c) shows the specific capacity of the pristine and composites calculated from the CD curves obtained at a current density of 0.5 A g^−1^ in a potential window range of 0.0–0.4 V. In Fig. 5(c), it is obvious that Ni_3_(PO_4_)_2_/90 mg GF composite shows the highest specific capacity of 48 mA h g^−1^, and this could be attributed to the fast charge transfer between the composite and the electrolyte.

**Fig. 6:**
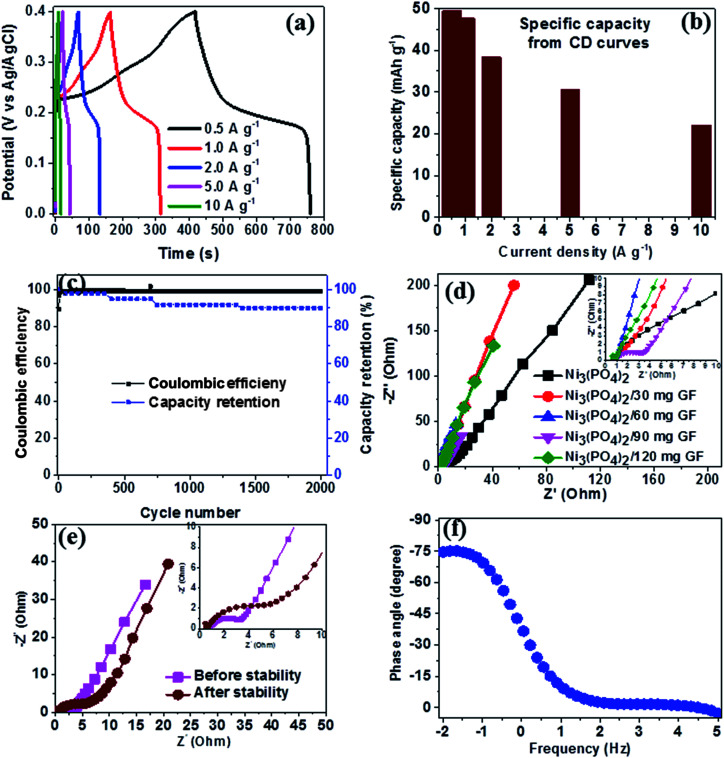
Ni_3_(PO_4_)_2_/90 mg GF composite electrochemical data: (a) CD curves at different current densities, (b) specific capacity as a function of current density, (c) the cycling stability at a current density of 10 A g^−1^, (d) Nyquist plots for the samples shown in the (the insert shows the enlarged high-frequency region of the plot), (e) Nyquist before and after stability test and (f) the phase angle as a function of frequency.

Fig. 6(a) shows the CD curves at current densities in the range of 0.5–10 A g^−1^ in the potential window range of 0.0–0.4 V for Ni_3_(PO_4_)_2_/90 mg GF composite. Clearly, the CD curves are nonlinear which further confirm the faradic property of the electrode material. The specific capacity of Ni_3_(PO_4_)_2_/90 mg GF composite was calculated using eqn (1) and the values are 48, 41, 37, 29 and 25 mA h g^−1^ for current densities of 0.5, 1.0, 2.0, 5.0 and 10.0 A g^−1^ respectively, as shown in Fig. 6(b). The long-time cyclic performance of Ni_3_(PO_4_)_2_/90 mg GF composite, which is an important parameter for supercapacitor practical applications is shown in Fig. 6(c). In Fig. 6(c), within the first 400 cycles, the specific capacity gradually increases mainly due to the activation of the electrode material. Thereafter, 92% of the initial specific capacity was considerably retained after 2000 cycles while coulombic efficiency remained at approximately 99%, implying that the faradaic redox response is nearly reversible.

In order to further assess the electrical resistance of Ni_3_(PO_4_)_2_ and Ni_3_(PO_4_)_2_/GF composites, the electrochemical impedance spectroscopy (EIS) at the potential of 0.0 V and the frequency range of 10–100 mHz was carried out. The Nyquist plots were obtained as shown in Fig. 6(d) for the Ni_3_(PO_4_)_2_ Ni_3_(PO_4_)_2_/30 mg GF, Ni_3_(PO_4_)_2_/60 mg GF, Ni_3_(PO_4_)_2_/90 mg GF and Ni_3_(PO_4_)_2_/120 mg GF samples. From this plot, two distinct behaviours for the electrode material can be seen at the high-frequency (the lower left portion of the curve) and low-frequency (the upper right portion of the curve) regions. The intercept of the EIS plot with the Z′-axis gives information about the equivalent series resistance (ESR) value of the electrode.^39^ This comprises the internal resistance of the electrode and electrolyte, plus the contact resistance at the active material/current collector interface. The ESR is also commonly referred to as the solution resistance (*R*_S_). From the inset to the figure, the intercept with the *x*-axis of the Ni_3_(PO_4_)_2_, Ni_3_(PO_4_)_2_/30 mg GF, Ni_3_(PO_4_)_2_/60 mg GF, Ni_3_(PO_4_)_2_/90 mg GF and Ni_3_(PO_4_)_2_/120 mg GF are 0.78, 0.73, 0.71, 0.68 and 0.78 Ω respectively which correspond to *R*_S_ values. In addition, the inclined region of the curve (low-frequency region) should in principle be a vertical line parallel to the *y*-axis, however, the inclination is due to the presence of a leakage resistance.^40^Fig. 6(e) shows the Nyquist plots of Ni_3_(PO_4_)_2_/90 mg GF composite before and after stability. The plots show almost a semi-circular arc in the high-frequency region as a result of the charging of the double layer which appears in the medium frequency region of the plot. This is attributed to the interfacial charge transfer resistance and mass transport through the material and is denoted by *R*_CT_. The other feature that is observed for this sample is shortest diffusion length as compared to the other samples (see Fig. 6(d)) which a clear indication of fast ion diffusion capability in the electrode. The *R*_S_ value of Ni_3_(PO_4_)_2_/90 mg GF composite before and after stability is 0.68 Ω which is the lowest compared to other electrode materials, while the *R*_CT_ values before and after stability were obtained to be 2.92 Ω and 6.22 Ω respectively. The material exhibited slightly higher charge transfer resistance after 2000 charge–discharge cycles which could be due to the decrease in electrical conductivity of the electrode material. Fig. 6(f) shows the phase angle as a function of frequency for Ni_3_(PO_4_)_2_/90 mg GF composite, and this presents the phase angle value of about −75° which is close to the ideal value of −90° suggesting that the electrode material approximates the ideal capacitive behavior.

Moreover, compared with the previous literature on electrochemical performances of nickel/metal phosphate-based electrodes evaluated in a three-electrode cell configuration, a Ni_3_(PO_4_)_2_/90 mg GF composite material display higher specific capacity and excellent rate capability as illustrated in Table 1.

**Table 1:** A comparison of electrochemical performances of nickel/metal phosphate-based electrodes evaluated in a three-electrode cell configuration found in literature and this work

Electrode materials	Synthesis method	Specific capacity (mA h g^−1^)	Electrolyte	Cyclic performance	Ref.
NaNiPO_4_	Hydrothermal	4.17 mA h g^−1^	2 M NaOH	99% after 2000 cycles@1.0 A g^−1^	20
Ni_11_(HPO_3_)_8_(OH)_6_	Hydrothermal	32@0.625 A g^−1^	3 M KOH	99.3% after 1000 cycles@0.625 A g^−1^	19
Co_2_P	Thermal decomposition	83@1.0 A g^−1^	3 M KOH	97% after 6000 cycles@5.0 A g^−1^	41
Mn_3_(PO_4_)_2_/GF	Hydrothermal	29@0.5 A g^−1^	6 M KOH	99% after 100 cycles@5.0 A g^−1^	25
Mn_3_(PO_4_)_2_	Hydrothermal	25@0.5 A g^−1^	2 M KOH	91% after 1000 cycles@1.0 A g^−1^	42
Mn_3_(PO_4_)_2_	Hydrothermal	41@0.5 A g^−1^	1 M Na_2_SO_4_	88.9% after 1000 cycles@1 A g^−1^	42
Ni_3_(PO_4_)_2_/90 mg GF	Hydrothermal	48@0.5 A g^−1^,	6 M KOH	99% columbic efficiency and 92% capacity retention after 2000 cycles@10 A g^−1^	This work

**Fig. 7:**
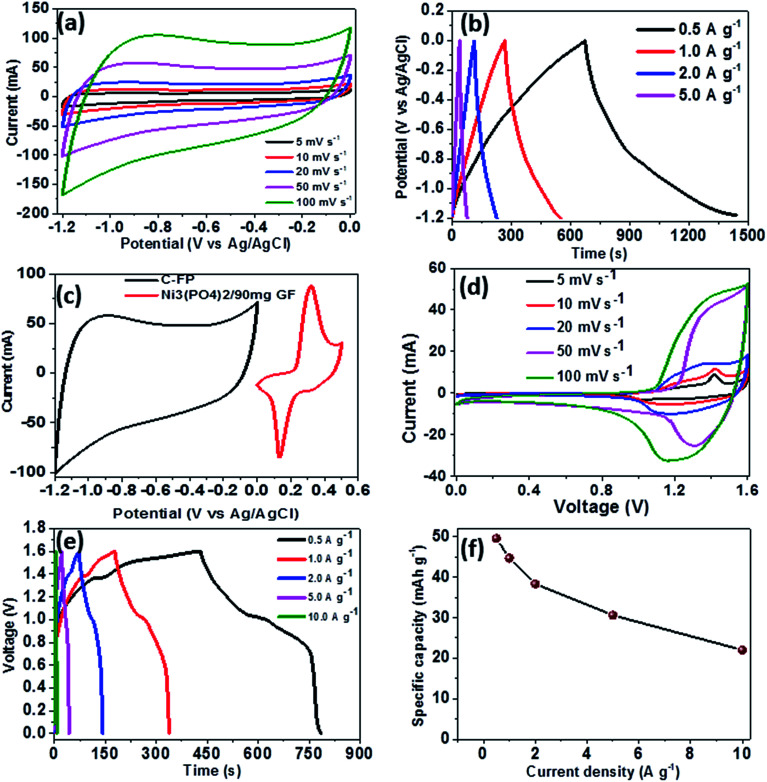
(a) CV curves of the C-FP electrode at different scan rates in a potential window range of −1.2 to 0.0 V, (b) CD curves of the C-FP electrode at different current densities in the range of 0.5–5.0 A g^−1^, (c) CV curves of the Ni_3_(PO_4_)_2_/90 mg GF and C-FP at a scan rate of 50 mV s^−1^, (d) CV curves of the Ni_3_(PO_4_)_2_/90 mg GF//C-FP hybrid device at scan rates of 5–100 mV s^−1^, (e) CD curves of the hybrid device at current densities of 0.5–10 A g^−1^ and (f) the specific capacity as a function of current density for the Ni_3_(PO_4_)_2_/90 mg GF//C-FP hybrid device.

To further investigate the electrochemical performance of Ni_3_(PO_4_)_2_/90 mg GF electrode material, a Ni_3_(PO_4_)_2_/90 mg GF//C-FP hybrid device was fabricated based on Ni_3_(PO_4_)_2_/90 mg GF and carbonized iron cations (Fe^3+^) adsorbed onto polyaniline (PANI) (C-FP) as positive and negative electrode respectively. This hybrid device configuration was adopted as it offers a wider operating potential window of 0.0–1.6 V, which would result in a high energy density. For a fabrication of hybrid supercapacitor device, the CV and CD curves of a C-FP negative electrode were recorded in a three-electrode cell configuration to determine the maximum working potential window of the device, as shown in Fig. 7(a) and (b) respectively. In Fig. 7(a), it can be observed that the CV curves of the C-FP electrode show ideal capacitive behavior with a quasi-rectangular shape in a potential window range of −1.2–0.0 V at the scan rates of 5–100 mV s^−1^. These CV curves indicate the capacitive behavior of a C-FP electrode. Fig. 7(b) shows the CD curves of the C-FP electrode at different current densities in the range of 0.5–5.0 A g^−1^. These CD curves are almost linear proposing that the electrode has behavior that in agreement with the CV curves. The additional results of the C-FP are presented in Fig. S3 to S5 (ESI).†

The amount of charge, *Q*, stored in each of the positive and negative electrodes in the hybrid device must be equal and is governed by the following equations:^43^

4a *Q*_+_ = *Q*_−_

4b *m*_+_*I*_d(+)_Δ*t*_+_ = *m*_−_*I*_d(−)_Δ*t*_−_

where *Q*_+_ (C) and *Q*_−_ (C) are charges stored on positive and negative electrodes respectively; *m*_+_ and *m*_−_ are the masses of the positive and negative electrodes respectively, *I*_d(+)_ and *I*_d(−)_ are the current density used in both positive and negative electrodes, Δ*t*_+_ and Δ*t*_−_ are discharge time in the positive and negative electrodes respectively.

According to the eqn (4a) and (4b), a hybrid asymmetric device designated as Ni_3_(PO_4_)_2_/90 mg GF//C-FP was fabricated with mass balance ratio estimated as 1.0: 0.54 corresponding to a loading mass of approximately 2.6 mg cm^−2^ and 1.4 mg cm^−2^ for both the positive (Ni_3_(PO_4_)_2_/90 mg GF) and negative (C-FP) electrodes respectively, giving the total mass loading of the active materials in the hybrid device as 4.0 mg cm^−2^.

Based on the synergy of potential window range of the C-FP (−1.2–0.0 V) and that of the Ni_3_(PO_4_)_2_/90 mg GF (0.0–0.4 V), as shown in Fig. 7(c), it can be observed that the fabricated Ni_3_(PO_4_)_2_/90 mg GF//C-FP hybrid device could operate in a much wider potential window of ∼1.6 V (Fig. 7(d)).

Fig. 7(d) shows the CV curves of the asymmetric Ni_3_(PO_4_)_2_/90 mg GF//C-FP hybrid device measured at various scan rates from 5 to 100 mV s^−1^. The CV curves at various scan rates can be observed to display a mixed electric-double layer capacitance and faradic behaviors which is a typical of hybrid asymmetric supercapacitor.^27^

Fig. 7(e) shows the non-linear CD curves of the hybrid device at different current densities, exhibiting a typical of faradic behaviour of the device. The specific capacities of the asymmetric device were calculated from the CD curves using eqn (1) and found to be approximately 48, 44, 38, 30, and 22 mA h g^−1^ at current densities of 0.5, 1.0, 2.0, 5.0, and 10 A g^−1^ respectively (Fig. 7(f)).

**Fig. 8:**
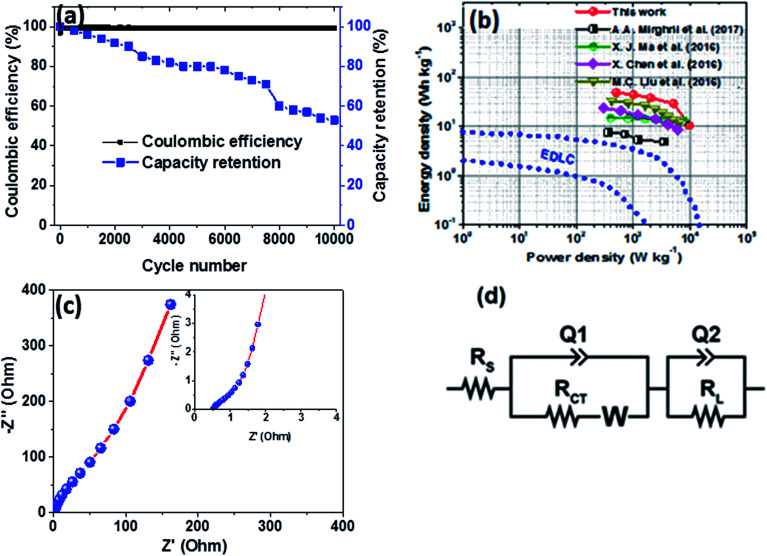
(a) Capacity retention and coulombic efficiency as a function of a cycle number for the Ni_3_(PO_4_)_2_/90 mg GF//C-FP hybrid device at a current density of 10 A g^−1^, (b) Ragone plot of the Ni_3_(PO_4_)_2_/90 mg GF//C-FP hybrid device compared to some other nickel/metal phosphate-based electrodes recently reported in the literature, (c) the Nyquist plot (the insert shows the enlarged high-frequency region of the plot) and (d) the equivalent circuit used for fitting the EIS data in (c) as shown by a solid line.

Furthermore, the hybrid device showed a good capacity retention and coulombic efficiency as a function of a cycle number as displayed in Fig. 8(a). Noticeably, in Fig. 8(a), initially the specific capacity increases up to 600 cycles and this might be due to the main activation process of the hybrid device. Thereafter, the specific capacity decreases gradually up to 53% over 10 000 cycles revealing a good electrochemical stability of the hybrid device and suggesting a superior cyclic performance for the electrode materials. Moreover, energy and power densities are important parameters to evaluate in the analysis of the electrochemical performance of the Ni_3_(PO_4_)_2_/90 mg GF//C-FP hybrid device. Therefore, Fig. 8(b) shows the Ragone plot of the device which describes the relationship between energy density (*E*_d_) and power density (*P*_d_) estimated according to eqn (2) and (3).

The hybrid device exhibited a maximum energy density of 49 W h kg^−1^ at a current density of 0.5 A g^−1^ with a corresponding power density of 499 W kg^−1^, and the energy density value decreases to 21.6 W h kg^−1^ at a current density of 10 A g^−1^ with a corresponding power density of 9720 W kg^−1^. In Fig. 8(b), both energy and power densities obtained for the hybrid device are much higher than those found in the literature for some recently published reports on nickel/metal phosphate-based electrodes evaluated in two-electrode cell devices.^41,42,44,45^ Besides, the energy densities obtained for the Ni_3_(PO_4_)_2_/90 mg GF//C-FP device fabricated in this work were observed to exhibit significant improvement compared to those of EDLC devices, A confirmation of the device's faradaic (battery-like) behaviour with such a high potential (1.6 V) in aqueous electrolyte.

Table 2 shows the electrochemical performance of the hybrid Ni_3_(PO_4_)_2_/90 mg GF//C-FP device compared to some other similar materials in the literature. It could be observed that the hybrid device exhibits considerable enhanced electrochemical performance compared to some other asymmetric devices in aqueous electrolyte (Table 2).

**Table 2:** A comparison of electrochemical performances of nickel/metal phosphate-based electrodes evaluated in two-electrode cell devices found in literature and this work

Electrodes	Energy density (W h kg^−1^)	Power density (W kg^−1^)	Cyclic performance	Ref.
Co_2_P//graphene	24	300	97% after 6000 cycles@0.4 A g^−1^	41
Mn_3_(PO_4_)_2_/GF//AC	7.6	3528	96% after 10 000 cycles@2.0 A g^−1^	25
Mn_3_(PO_4_)_2_//AC	16.64	399.36	90% after 10 000 cycles@1.0 A g^−1^	42
Mn_3_(PO_4_)_2_//Mn_3_(PO_4_)_2_	19.09	392.78	90% after 10 000 cycles@1.0 A g^−1^	42
Ni_2_P_2_O_7_//graphene	23.4	1292.2	98.5% after 5000 cycles@1.0 A g^−1^	45
Ni_3_(PO_4_)_2_/90 mg GF//C-FP	49	499	98% after 10 000 cycles@10 A g^−1^	This work

To evaluate the conductivity and charge transport properties at the electrode/electrolyte interface, an electrochemical impedance spectroscopy (EIS) of the hybrid device was carried out (Fig. 8(c)). The EIS curve of Ni_3_(PO_4_)_2_/90 mg GF//C-FP device show almost unnoticed semi-circle in the high-frequency region followed by a linear component in the low-frequency region. Fig. 8(d) shows a circuit diagram used for fitting the EIS data (solid-lines in Fig. 8(c)). The circuit diagram presents the electronic resistance of the device, also known as the equivalent series resistance (*R*_S_) in series with the charge transfer resistance (*R*_CT_) at the high-frequency region and Warburg impedance characteristic element (*W*), which can be expressed as *A*/(*jω*)^0.5^, where *A* is the Warburg coefficient, *ω* is the angular frequency which is in parallel with the real capacitance (*Q*_1_). In the low-frequency region, an ideal electrode should be a vertical line parallel to the imaginary axis with a mass capacitance (*Q*_2_). The deviation from this ideal behaviour is usually attributed to a leakage resistance *R*_L_ arising from the faradaic charge transfer process, and this is always in parallel to the *Q*_2_ as shown in the equivalent circuit.^25,44,45^ The *R*_S_ value which represents the ohmic resistance of the materials was found to be 0.51 Ω while the *R*_CT_ which represents the charge-transfer kinetics and fast ion transport was found to be 0.89 Ω. These *R*_S_ and *R*_CT_ values are indicating fast ion diffusion and low charge transfer resistance suggesting an ideal capacitive performance of the materials.

## 4. Conclusions

A pristine Ni_3_(PO_4_)_2_ nano-rods and Ni_3_(PO_4_)_2_/GF composite samples with different GF mass loading were synthesised using a hydrothermal method. The structural, composition, and morphological properties of the pristine and composite samples were confirmed by XRD, Raman spectroscopy, XPS, SEM and HRTEM analysis. It has been observed from the structure and electrochemical performance of the composites that a variation in GF content of the Ni_3_(PO_4_)_2_/GF composite samples influences the synergy between the Ni_3_(PO_4_)_2_ and the GF. The specific capacity of the Ni_3_(PO_4_)_2_/GF composite samples was found to increase with increasing GF mass loading up to 90 mg, and thereafter, decreases as GF increases up to 120 mg. The Ni_3_(PO_4_)_2_/90 mg GF composite sample exhibited the highest specific capacity of 48 mA h g^−1^ at a current density of 0.5 A g^−1^ in 6 M KOH electrolyte. This value shows an excellent improvement from that of the pristine Ni_3_(PO_4_)_2_ sample which exhibited the specific capacity of 17 mA h g^−1^ at a current density of 0.5 A g^−1^. This improvement is attributed to the synergetic effect between the Ni_3_(PO_4_)_2_ and the GF. Moreover, the fabricated Ni_3_(PO_4_)_2_/90 mg GF//C-FP hybrid device was able to store a high energy and power densities of 49 W h kg^−1^ and 499 W kg^−1^ respectively at 0.5 A g^−1^ which was attributed to the combination of Ni_3_(PO_4_)_2_/90 mg GF with C-FP electrode material. In addition, the device showed a remarkable high power density of 9720 W kg^−1^ with corresponding energy density of 21 W h kg^−1^ and long-term stability, which retained 53% of the initial capacity after 10 000 cycles at high current density of 10 A g^−1^.

## Conflicts of interest

There are no conflicts to declare.

## Supplementary Material

RA-008-C7RA12028A-s001
