# Correction: A high energy density asymmetric supercapacitor utilizing a nickel phosphate/graphene foam composite as the cathode and carbonized iron cations adsorbed onto polyaniline as the anode

**DOI:** 10.1039/c8ra90028k

**Published:** 2018-04-17

**Authors:** A. A. Mirghni, M. J. Madito, K. O. Oyedotun, T. M. Masikhwa, N. M. Ndiaye, Sekhar C. Ray, N. Manyala

**Affiliations:** Department of Physics, Institute of Applied Materials, SARCHI Chair in Carbon Technology and Materials, University of Pretoria Pretoria 0028 South Africa ncholu.manyala@up.ac.za +(27)12 420 2516 +(27)12 420 3549; Department of Physics, College of Science, Engineering and Technology, University of South Africa Private Bag X6, Florida, 1710, Science Campus, Christiaan de Wet and Pioneer Avenue, Florida Park Johannesburg 1710 South Africa

## Abstract

Correction for ‘A high energy density asymmetric supercapacitor utilizing a nickel phosphate/graphene foam composite as the cathode and carbonized iron cations adsorbed onto polyaniline as the anode’ by A. A. Mirghni *et al.*, *RSC Adv.*, 2018, **8**, 11608–11621.

Sekhar C. Ray was incorrectly spelled in the published article; the corrected version is shown above.

The Royal Society of Chemistry apologises for these errors and any consequent inconvenience to authors and readers.

## Supplementary Material

