# Supplementary material for: A high energy density asymmetric supercapacitor utilizing a nickel phosphate/graphene foam composite as the cathode and carbonized iron cations adsorbed onto polyaniline as the anode
Source: RSC Adv. 2018 Mar 26;8(21):11608–21. doi: 10.1039/c7ra12028a (PMC9079250; doi:10.1039/c7ra12028a)
Supplement: RA-008-C7RA12028A-s001 [file RA-008-C7RA12028A-s001.pdf]

Electronic Supplementary Material (ESI) for RSC Advances.
This journal is © The Royal Society of Chemistry 2019

High energy density asymmetric supercapacitor utilizing nickel phosphate/graphene foam composite as cathode and carbonized iron cations adsorbed onto polyaniline as anode

A. A. Mirghni [a], M. J. Madito [a], K. O. Oyedotun [a], T. M. Masikhwa [a], N. M. Ndiaye [a], S. C. Ray [b] and N. Manyala [a]*

[a] Department of Physics, Institute of Applied Materials, SARCHI Chair in Carbon Technology and Materials, University of Pretoria, Pretoria 0028, South Africa.

[b] Department of Physics, College of Science, Engineering and Technology, University of South Africa, Private Bag X6, Florida, 1710, Science Campus, Christiaan de Wet and Pioneer Avenue, Florida Park, Johannesburg 1710, South Africa

*Corresponding author's email: ncholu.manyala@up.ac.za, Tel.: + (27)12 420 3549; Fax: + (27)12 420 2516

Supporting Information

To obtain the thickness (number of layers) of the GF, the as-prepared GF sheet was investigated using HRTEM and the HRTEM micrograph of a GF sheet is shown in Fig. S1(a) along with the corresponding selected area electron diffraction (SAED) pattern (Fig. S1(b)). The HRTEM micrograph of GF displays folded area showing fringes (2-folds) corresponding to two layers of graphene which has a distinctively selected area electron diffraction pattern (SAED) of a graphene, as shown in Fig. S1(b). This suggests that a GF used in this work is a few-layered graphene with layers number approximately less than five, and it is high- quality graphene.

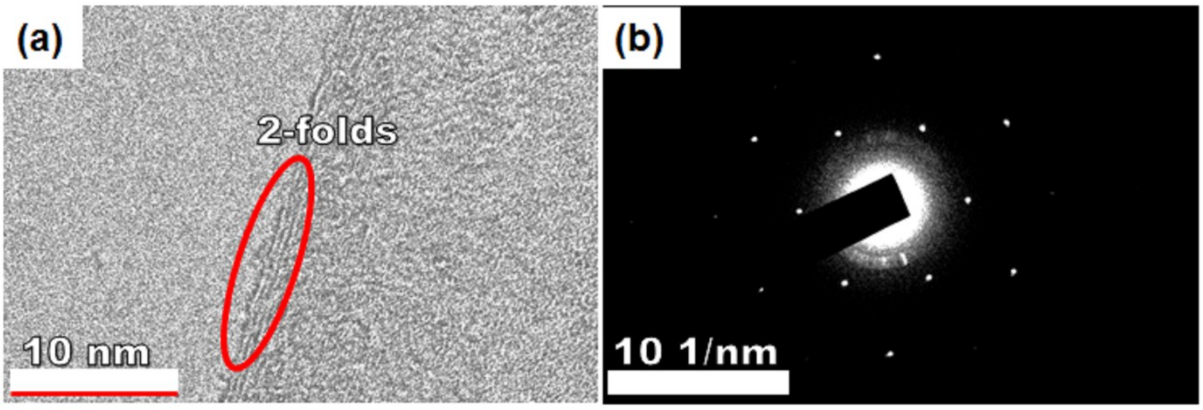

Figure S1. (a) An HRTEM image of the GF and (b) the corresponding SAED image.

Fig. S2(a) shows the CV curves of pristine $Ni_3(PO_4)_2$ at different scan rates (10–100 mV s^{-1}) in a potential window range of 0.0–0.5 V. In Fig. S2(a), an observed oxidation and reduction peaks around 0.16 V and 0.34 V correspond to anodic and cathodic peaks respectively, these peaks reveal the faradic behavior of the pristine $Ni_3(PO_4)_2$ electrode material. It can also be observed that the intensity or rather the current of the anodic and cathodic peaks is almost the same which implies that the redox reaction is reversible. Consequently, the anodic and cathodic peaks can be ascribed to the extraction and reversible intercalation of potassium ions into/from the $Ni_3(PO_4)_2$ resulting in Ni^{3+} and Ni^{2+} ions of oxidation and reduction states. Fig. S2(b) shows the CD curves of pristine $Ni_3(PO_4)_2$ electrode at different current densities (i.e., 0.5–10 A g^{-1}) in a potential window range of 0.0–0.4 V. The CD curves of the $Ni_3(PO_4)_2$ electrode show potential steps (i.e., fast drop in a potential range of 0.4–0.25 V and slow drop in a potential range of 0.16–0.22 V) suggesting faradic behavior of the pristine $Ni_3(PO_4)_2$ electrode material. It is worth mentioning that the $Ni_3(PO_4)_2$ material has a potential window range of 0.0–0.4 V as shown in CD curves in Fig. S2(b), however, it is evaluated in a potential window range of 0.0–0.5 V in CV curves to obtain a clear peak-shape of a cathodic peak at about 0.34 V.

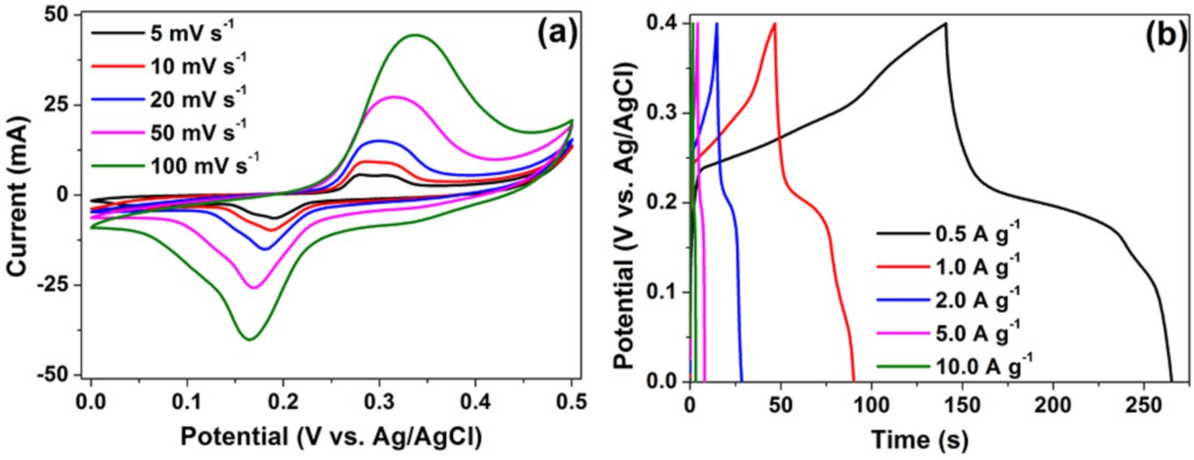

Figure S2: (a) CV curves of pristine $Ni_3(PO_4)_2$ at different scan rates (10–100 mV s^{-1}) in a potential window range of 0.0–0.5 V and (b) CD curves of pristine $Ni_3(PO_4)_2$ at different current densities.

Figure S3(a and b) shows the SEM morphology of the carbonized iron cations (Fe^{3+}) adsorbed onto polyaniline (PANI) (C-FP) at low and high magnification which shows the agglomerated porous nanograin particles of the C-FP.

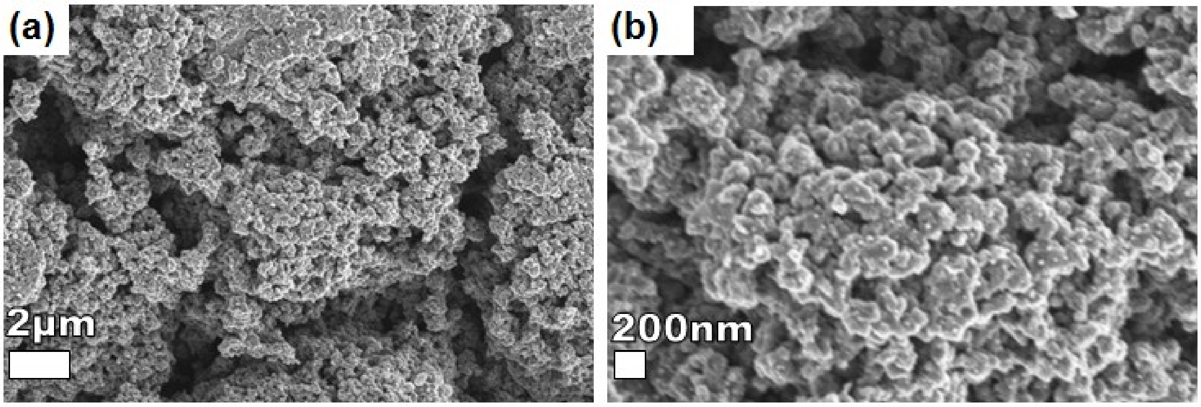

Figure S3. (a and b) SEM micrographs of the carbonized iron cations (Fe^{3+}) adsorbed onto PANI (C-FP) at low and high magnification.

Figure S4 shows the XRD of the C-FP sample indexed using the matching Inorganic Crystal Structure Data-base (ICSD) card no. 16593 with chemical formula Fe_3C, crystal system: orthorhombic and space-group: *P n m a*. The XRD pattern shows a significant amount of

orthorhombic Fe_3C predominantly due to the dissolution of carbon atoms into Fe lattices during pyrolysis at a high temperature of about 850 °C. A diffraction peak at 52.4° is from both metallic Fe and Ni, in the case of Fe, during the pyrolysis process, Fe^{3+} could be reduced to metallic iron by the reaction with pyrolytic carbon from PANI. An expected broad and weak diffraction peak around 30° that could be assigned to the XRD graphitic plane (002), indicating a graphitization of carbon material is suppressed by high-intensity peaks of Ni substrate.

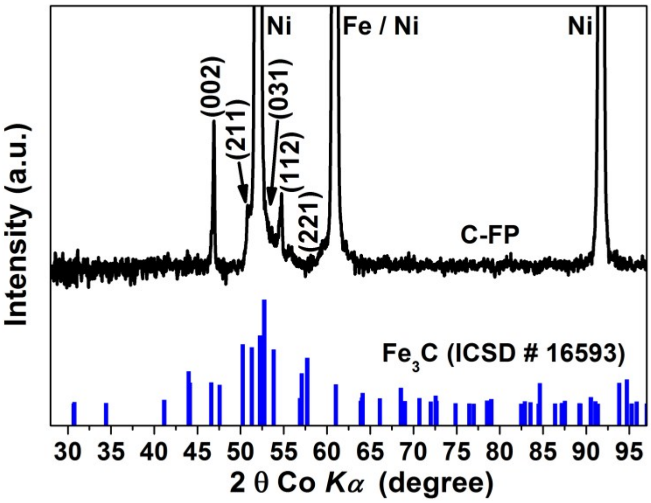

Figure S4. The XRD of the carbonized iron cations ($Fe^{3+)}$ adsorbed onto PANI (C-FP).

Figure S5(a) shows the specific capacitance of the C-FP electrode as a function of current density which was calculated from the CD curves as 321, 235, 190 and 158 F g^{-1} at current densities of 0.5, 1.0, 2.0 and 5.0 A g^{-1} respectively. Figure S5(b) shows the Nyquist impedance plot of the C-FP electrode, and the insert shows the enlarged high-frequency region of the plot which clearly shows the semicircle attributed to the interfacial charge transfer resistance and mass transport through the material.

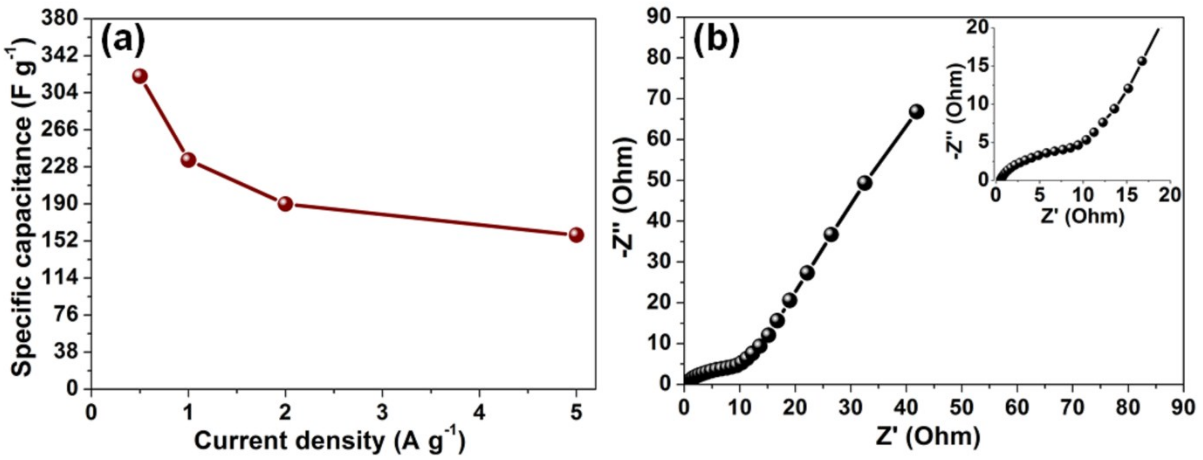

Figure S5. (a) The specific capacitance of the C-FP as a function of current density (b) Nyquist plot (imaginary component, Z" versus the real component, Z' of the impedance) of the C-FP (the insert shows the enlarged high-frequency region of the plot)
